# California State Veterinary Medical Association

**Published:** 1894-10

**Authors:** R. A. Archibald

**Affiliations:** Secretary; Sacramento, Cal.


### CALIFORNIA STATE VETERINARY MEDICAL ASSOCIATION.

A regular meeting of the California State Veterinary Medical Association was held on June 13th, at the Baldwin Hotel, San Francisco, Cal.

The meeting was called to order by the President, Dr. H. A. Spencer.

Upon roll call, the following gentlemen answered to their names: Drs. Whittlesey, Fox, Spencer, Sr., Spencer, Jr., Forrest, Egan, Robin, Williams, Hoggarty, Pierce, Eddy, Maclay, Jackson, Orvis and Archibald; visitor, Dr. T. T. Twombly, Logan, Utah. Under the head of Reports of the Boards of Examiners, Committees, etc., the Board of Examiners reported favorably upon the name of F. Forrest, and recommended that he be admitted to membership. The Committee on Certificates made their report, which was accepted, upon motion, and the committee discharged. The Committee on Legislative Matters reported that they had attended the State Sanitary Convention, held in San Jose, and they expressed the belief that they had accomplished a great deal towards the elevation of the veterinary profession by so doing; they also stated that they were treated very courteously, on that occasion, by the members of the medical profession who were present at the Convention.

Under the head of Admission of New Members, the Secretary moved that Dr. H. Forrest, of San Jose, be admitted to become a member of the Association; the motion was seconded by Dr. Fox, and carried.

Under the head of Reading of Papers, Discussions, etc., Dr. D. F. Fox, of Sacramento, read a very original and instructive paper on "Diseases of the Liver as a Direct Cause of Intestinal Disturbances." (See p. 340.) The President called upon Dr. Spencer, Jr., to open the discussion, which he did, by saying that he had been called upon to treat a great many cases such as described by the essayist, and he fully concurred with the essayist, in that the primary lesions in the sex cases are to be found in the hepatic gland, in the majority of cases. The discussion was also participated in by Drs Orvis, Whittlesey, Maclay, Twombly, Archibald and Egan. They all complimented the essayist on the originality of his remarks. Some of the members, however, did not agree with the essayist, and stated that it was their opinion that if there was liver disease present in these cases, it was purely a secondary lesion. Dr. Egan mentioned a very interesting case in which the cause of death was due to hepatic lesions. Dr. Spencer, Jr., said that he had treated the disease with aloin, followed by tonics, with good success. The Secretary stated that he had treated these cases with drastic purgatives without success, but since he had applied treatment toward the amelioration of hepatic derangements, he had quite a decrease in the mortality of the cases he was called upon to treat.

Dr. Whittlesey said he had held post-mortems on several of these cases without discovering any changes in the hepatic gland.

The President closed the discussion with a few well-chosen remarks. He said this was a subject which should receive a great deal of attention at our hands; he believed that there were a great many cases of intestinal disturbances due to hepatic lesions. In closing his remarks, he stated that he thought that we were, as an Association, very fortunate in having a membership of gentlemen who were particularly desirous of introducing at our meetings subjects of which very little was known, in order that in the exchange of ideas which follow the reading of the papers, points would be raised which would lead us, or enable us, to treat these cases on a more scientific basis.

Dr. J. H Eddy, of Stockton, was then called upon to entertain the meeting, which he did by reading a very practical thesis on " Tetanus." It was followed by an animated and lengthy discussion, which was confined principally to the etiology of the disease. The following gentlemen participated in the discussion: Drs Spencer, Jr., Whittlesey, Archibald, Orvis, Fox and Pierce.

Dr. Forrest was called upon to read a paper on " Springhalt." The essayist, without going into the pathology of the disease, described a case which he had treated, and in which he performed a cure by performing the operation known as pereneo prephalangeal tenotomy. The discussion that followed was participated in by Drs. Maclay, Whittlesey, Spencer, Jr., Twombly, Archibald and Egan.

The President closed the discussion with a few appropriate remarks.

On motion by Dr. Whittlesey, a vote of thanks was tendered the essayists for the able and masterly manner in which they had entertained the meeting.

Dr. Twombly was requested to give an account of a few experiments he had made with tuberculin, in Utah, a request which he cheerfully complied with.

The Secretary proposed the name of J. H. Edmons, of Los Angeles, for membership. The name was referred to the Board of Examiners.

On motion by the Secretary, the time of meeting was changed from 7.30 P. M. to 2 P M., in order to accommodate the country members.

The Secretary gave notice that at the next quarterly meeting he would move to amend the By-Laws regarding the place of meeting. The matter was referred to the Board of Directors.

The President appointed the following named gentlemen as essayists for the next meeting: Drs. Egan, Skaife and Hoggarty.

The following bills were ordered paid: R. A. Archibald, $8.75, for Secretary's supplies; J. J. Evans, $15.00, for certificates; J. A. Cowen, $60.00, for printing pamphlets.

The Secretary presented a communication from the Secretary of the United States Veterinary Medical Association, regarding the possibility of having some representation at the next meeting of the national organization.

Dr. Robin spoke on the use of vucatol in the treatment of fistulæ, cancers, etc., and he presented each member with a sample package.

There being no further business to come before the meeting, it adjourned, to meet in San Francisco, September 12th, 1894.

R. A. ARCHIBALD, D. V. S.,
*Secretary.*

SACRAMENTO, CAL.

